# A Detailed Study on Understanding Glycopolymer Library and Con A Interactions

**DOI:** 10.1002/pola.26646

**Published:** 2013-03-13

**Authors:** Yanzi Gou, Jin Geng, Sarah-Jane Richards, James Burns, C Remzi Becer, D M Haddleton

**Affiliations:** 1 Science and Technology on Advanced Ceramic Fibers and Composites Laboratory, National University of Defense TechnologyChangsha, 410073, China; 2 Faculty of Engineering, University of BristolClifton BS8 1TR, United Kingdom; 3 Department of Chemistry, University of WarwickCoventry CV4 7AL, United Kingdom

**Keywords:** biomaterials, radical polymerization, synthesis

## Abstract

Synthetic glycopolymers are important natural oligosaccharides mimics for many biological applications. To develop glycopolymeric drugs and therapeutic agents, factors that control the receptor-ligand interaction need to be investigated. A library of well-defined glycopolymers has been prepared by the combination of copper mediated living radical polymerization and CuAAC click reaction via post-functionalization of alkyne-containing precursor polymers with different sugar azides. Employing Concanavalin A as the model receptor, we explored the influence of the nature and densities of different sugars residues (mannose, galactose, and glucose) on the stoichiometry of the cluster, the rate of the cluster formation, the inhibitory potency of the glycopolymers, and the stability of the turbidity through quantitative precipitation assays, turbidimetry assays, inhibitory potency assays, and reversal aggregation assays. The diversities of binding properties contributed by different clustering parameters will make it possible to define the structures of the multivalent ligands and densities of binding epitopes tailor-made for specific functions in the lectin-ligand interaction. © 2013 Wiley Periodicals, Inc. J. Polym. Sci., Part A: Polym. Chem. 2013, 51, 2588–2597

## INTRODUCTION

Synthetic glycopolymers containing pendant saccharide moieties have been employed as multivalent natural oligosaccharide mimics in many biological and biomedical applications such as macromolecular drugs and drug delivery systems.^1^–^6^ To develop novel glycopolymeric drugs and therapeutic agents, numerous efforts have been devoted to the synthesis of glycopolymers.^7^–^12^ With the development of synthetic technologies in recent years, it is now desirable to prepare well-defined glycopolymers either by polymerization of glycomonomers^13^–^17^ or by post-functionalization of precursor polymers.^18^–^20^ Kiessling and coworkers synthesized a series of glycopolymers by ring-opening metathesis polymerization (ROMP) using mannose- and galactose-substituted monomers.^21^ While Stenzel et al. employed radical addition-fragmentation chain-transfer polymerization to prepare glycopolymers from a glucose-substituted glycomonomer.^22^ However, direct polymerization often needs additional steps for synthesis and purification of glycomonomers, which have tendency to self-polymerization. In this case, the post-functionalization strategy shows a promising way to the synthesis of glycopolymers. In our group, we employed a facile approach to synthesize glycopolymers via the combination of copper mediated living radical polymerization (often called ATRP) and Huisgen copper(I)-catalyzed azide–alkyne cycloaddition (CuAAC). Alkyne-containing polymer scaffolds were prepared followed by modification of the polymer scaffolds with sugar azides.^23^–^26^

Besides syntheses, the investigation into lectin-glycopolymer interactions is also vital for the application of the relevant glycopolymers. The potencies of glycopolymers acting as inhibitors or effectors in biological applications depend on the specific mechanism by which they operate.^2^,^27^ These mechanisms can be the chelate effect, steric stabilization, substrate binding, receptor clustering, and statistical rebinding, etc.^28^ The macromolecular features (polymer architecture, chain length, polydispersity, and epitope density) determine the binding efficiency and define the function of the glycopolymers.^19^,^21^ Although some modern techniques such as quartz crystal microbalance^29^,^30^ and surface plasmon resonance,^3^,^31^,^32^ have been employed to explore the lectin-glycopolymer interactions, the traditionally used methods (quantitative precipitation, turbidimetry, fluorescence quenching assays, etc.) provide straightforward and important information about the inhibition and clustering of receptors.^33^–^36^

Kiessling and coworkers^21^,^28^ showed that factors such as glycopolymeric architecture, valency, size, and density of binding elements influenced the clustering of model receptor, Concanavalin A (Con A). The stoichiometry of the glycopolymer-Con A conjugates, the rate of ligands-lectin aggregation and the average inter receptor distances depended on the macromolecular properties of the multivalent ligands. By comparing with the ligands of low molecular weight, dendrimers, glycoproteins, and polydisperse glycopolymers with very high molecular weight, they indicated that the linear glycopolymers generated by ROMP were especially effective in the receptor clustering. Preliminary study^25^ of the lectin-glycopolymer interaction in our group also showed some results very interesting. These linear glycopolymers with different epitope densities showed excellent clustering properties to the lectin Con A.

**FIGURE 1:**
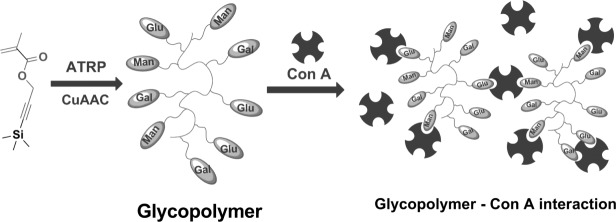
Schematic representation of the glycopolymer lectin binding.

Therefore, in this study similar linear glycopolymers were synthesized, which have the same macromolecular features differing only in the nature and proportions of the pendant binding units. The binding properties of these multivalent ligands introduced by not only the epitope densities but also the different nature of sugars are investigated, Figure 1.

## EXPERIMENTAL

### Materials

Copper(I) bromide, tetrabutylammonium fluoride (TBAF) in THF, acetic acid, (1.0 M), 2,2′-bipyridyl (bipyridine), 2-chloro-1,3-dimethylimidazolinium chloride, sodium azide, and Con A were purchased from Sigma-Aldrich. Triethylamine was purchased and used directly from Fisher Scientific. 3-(Trimethylsilyl)−2-propyn-1-ol, d-(+)-galactose and d-(+)-mannose were purchased from Alfa-Aesar. Copper(I) bromide was purified according to the method of Keller and Wycoff.^37^ 2-Bromo-2-methyl-propionic acid benzyl ester initiator,^38^ TMS-protected propargyl methacrylate^25^ and the ligands *N*-ethyl-2-pyridylmethanimine^39^ were prepared as described previously. HBS buffer (0.10 M HEPES, 0.9 M NaCl, pH 7.4) containing 1 mM metal ions (Ca^2+^, Mg^2+^, and Mn^2+^) was prepared using Milli-Q water as the buffer solution for all the interaction experiments. All other reagents and solvents were purchased at the highest purity available from Sigma-Aldrich Chemical Company and used without further purification unless stated.

### Characterization

All polymerizations were carried out using standard Schlenk techniques under an inert atmosphere of oxygen-free nitrogen, unless otherwise stated. ^1^H and ^13^C NMR spectra were obtained on a Bruker DPX-400 and Bruker DPX-300 spectrometer. All chemical shifts (^1^H and ^13^C) are reported in ppm (*δ*) relative to tetramethylsilane (TMS), referenced to the chemical shifts of deuterated solvent from Sigma-Aldrich. The following abbreviations were used to explain the multiplicities: d = doublet, m = multiplet, t = triplet. The molecular weight of the polymers *M*_n_ (NMR) was calculated by comparing the integrals of the benzyl chain-end signals and appropriate peaks related to the polymer backbone. FTIR spectra were recorded on a Bruker Vector-22 FTIR spectrometer using a Golden Gate diamond attenuated total reflection cell. Molecular weight and polydispersity of glycopolymers were measured using size exclusion chromatography (SEC) on a Varian 390-LC system in *N,N*-dimethylformamide (DMF; 1 g/L LiBr) at 50 °C, equipped with refractive index and viscometry detectors, 2 × PL gel 5 μm mixed D columns (300 × 7.5 mm), 1 × PLgel 5 μm guard column (50 × 7.5 mm) and autosampler. Data was analyzed using Cirrus 3.2 software. Molecular weight was determined relative to narrow poly(methyl methacrylate) standards. While molar mass distributions of the precursor polymer and the clickable polymer were measured on a system in chloroform/triethylamine (95:5 v/v, 1.0 mL min^−1^) equipped with two PL gel 5 µm mixed D columns (300 × 7.5 mm) and one PL gel 5 µm guard column (50 × 7.5 mm; 200–400,000 g/mol) with a differential refractive index detector calibrated with linear poly(methyl methacrylate) standards. All of the UV data was performed on a Varian Cary 50 Bio UV–vis spectrometer, using 2 mL volume polycarbonate cuvettes (1 cm path length).

### Synthesis of TMS-Protected Polymers

TMS-protected propargyl methacrylate (2.00 g, 10.2 mmol), *N*-(ethyl)−2-pyridylmethanimine ligand (0.078 mL, 0.51 mmol), initiator (0.094 g, 0.26 mmol), and mesitylene (0.5 mL) and toluene (8.0 mL) were charged into a dry Schlenk tube. After five freeze-pump-thaw cycles, the solution was then transferred under nitrogen into a second Schlenk tube, which was previously evacuated and filled with nitrogen containing Cu(I)Br (0.036 g, 0.25 mmol). The solution was stirred at 70 °C and samples were taken out periodically using a degassed syringe. At the end of the reaction, the mixture was diluted with 20 mL of toluene and then bubbled through with air for 4 h. The solution was passed through a short neutral alumina column and sequentially washed with toluene. The volatiles were removed under reduced pressure and the residues were dissolved in THF (ca. 10 mL) prior to precipitation into methanol/water (10:2 vol/vol) mixture (ca. 200 mL). The white solid was isolated by filtration, washed with additional methanol/water mixture, and volatiles removed under reduced pressure.

### Deprotection of the TMS-Protected Polymers

The TMS-protected polymer (1.5 g, 7.653 mmol alkyne-trimethylsilyl groups) and acetic acid (2.19 mL, 0.0382 mol, 5 equiv. to the alkyne-trimethylsilyl groups) were dissolved in THF (100 mL). Nitrogen was bubbled (ca. 10 min) and the solution was cooled to −20 °C. A 0.20 M solution of TBAF·3H_2_O (0.0114 mol, 1.5 equiv. to the alkyne-trimethylsilyl groups) was added slowly into the reaction mixture in ca. 20 min. The resulting mixture was stirred at this temperature for 30 min and then warmed to ambient temperature. After stirring overnight Amberlite IR-120 (PLUS) ion-exchange resin was added and stirred with the solution for 30 min. The resin was then removed by filtration under gravity and the resulting solution was concentrated under reduced pressure. The clickable polymer was isolated by precipitation in petroleum ether as white powder.

### Synthesis of Sugar Azides

The experiments followed a procedure as previously described.^24^–^26^

#### 2′-Azidoethyl-O-α-k-glucopyranoside

^1^H NMR (400.03 MHz, D_2_O, 298 K) *δ* = 3.32, 3.52 (m, 2H, CH_2_N_3_); 3.57–3.61 (m, 2H, C*H*_2_CH_2_N_3_); 3.61–3.69 (m, 2H, C*H_2_*OH); 3.71–3.97, 3.97–4.14 (m, 4H, 4×CH); 4.57 (d, *J* = 7.92 Hz, 1H, C_anomeric_H). 5.03 (d, *J* = 3.65 Hz, 1H, C_anomeric_H). ^13^C{^1^H} NMR (100.39 MHz, D_2_O, 298 K) *δ* = 50.45, 50.62 (1C, CH_2_N_3_); 60.59, 60.78 (1C, CH_2_OH); 66.30, 68.57 (1C, CH_2_*C*H_2_O); 69.56, 69.65 (1C, CH); 71.24, 72.01 (1C, CH); 72.95, 73.13 (1C, CH); 75.72, 75.97 (1C, CH); 98.29 (C_anomeric_); 102.33 (C_anomeric_); FTIR (neat):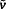 = 3358 (bs), 2927, 2097, 1644, 1301, 1262, 1132, 1056, 976, 913, 881, 812 cm^−1^ Anal. Calcd. for C_8_H_15_N_3_O_6_ C, 38.55; H, 6.07; N, 16.86; Found: C, 38.45; H, 6.07; N, 16.75; Mass Spectrometry (+ESI-MS) *m/z* (%): 102 (100), 272 [M + Na] (39).

#### 2′-Azidoethyl-O-α-d-mannopyranoside

^1^H NMR (400.03 MHz, D_2_O, 298 K) *δ* = 3.45 (m, 2H, CH_2_N_3_); 3.55–3.60 (m, 2H, C*H*_2_CH_2_N_3_); 3.61–3.67 (m, 2H, C*H_2_*OH); 3.67–3.91 (m, 4H, 4×CH); 4.92 (d, *J* = 1. 5 Hz, 1H, CH). ^13^C{^1^H} NMR (100.59 MHz, D_2_O, 298 K) *δ* = 50.20 (1C, CH_2_N_3_); 60.91 (1C, CH_2_OH); 66.30 (1C, CH_2_*C*H_2_O); 66.68 (1C, CH); 69.97 (1C, CH); 70.39 (1C, CH); 72.89 (1C, CH); 99.81 (C_anomeric_); IR (neat):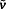 = 3358 (bs), 2927, 2097, 1644, 1301, 1262, 1132, 1056, 976, 913, 881, 812 cm^−1^; Anal. Calcd. for C_8_H_15_N_3_O_6_ C, 38.55; H, 6.07; N, 16.86; Found: C, 38.35; H, 6.11; N, 16.76; Mass Spectrometry (+ESI-MS) *m/z* (%): 102 (100), 118 (63), 172 (46), 217 (60), 272 [M + Na] (39).

#### 2′-Azidoethyl-O-β-D-galactopyranoside

^1^H NMR (400.03 MHz, D_2_O, 298 K) *δ* = 3.57 (m, 2H, CH_2_N_3_); 3.60 (m, 1H, CH) 3.64–3.72 (m, 2H, C*H*_2_CH_2_N_3_); 3.76–3.80 (m, 2H, C*H_2_*OH); 3.83 (m, 1H, CH); 3.93 (m, 1H, CH); 4.05 (m, 1H, CH); 4.46 (d, *J* = 7.78Hz, 1H, CH). ^13^C NMR (100.59 MHz, D_2_O, 298 K) *δ* = 50.55 (1C, CH_2_N_3_); 60.95 (1C, CH_2_OH); 68.38 (1C, CH_2_*C*H_2_O); 68.63 (1C, CH); 70.69 (1C, CH); 72.71 (1C, CH); 75.18 (1C, CH); 102.89 (C_anomeric_); IR (neat):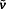 = 3322, 2953, 2098, 1644, 1303, 1265, 1121, 1061, 998, 910 cm^−1^ Anal. Calcd. for C_8_H_15_N_3_O_6_ C, 38.55; H, 6.07; N, 16.86; Found: C, 38.62; H, 6.00; N, 16.73; Mass Spectrometry (+ESI-MS) *m/z* (%): 102 (100), 118 (34), 172 (42), 217 (11), 272 [M + Na] (65).

#### α-Azido-d-mannose

^1^H NMR (400.03 MHz, D_2_O, 298 κ) *δ* = 3.66 (m, 1H, C*H*CH_2_); 3.72 (m, 1H, CH); 3.76 (m, 2H, CH_2_); 3.87 (m, 1H, CH); 3.95 (m, 1H, CH); 5.46 (d, *J* = 1.8 Hz, 1H, CH). ^13^C{^1^H} NMR (100.59 MHz, D_2_O, 298 K) *δ* = 61.46 (1C, CH_2_); 67.04 (1C, CH); 70.40 (1C, CH); 70.47 (1C, CH); 75.28 (1C, C*H*CH_2_); 90.38 (1C, C*H*N_3_). FTIR (neat):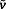 = 3313, 2111, 1238, 1062, 936, 805, 668, 587 cm^−1^. Anal. Calcd. for C_12_H_21_N_3_O_10_ C, 39.24; H, 5.76; N, 11.44; Found: C, 39.01; H, 5.84; N, 11.22. MS(ESI):105 (32) 129 (67) 157 (36) 185 (45) 228 (M + Na) (100).

### Synthesis of Glycopolymers by CuAAC

A solution of the clickable polymer (0.012 g, 0.092 mmol of “clickable” alkyne units), 2′-azidoethyl-O-α-d-mannopyranoside (0.026 g, 0.097 mmol), 2′-azidoethyl-O-β-D-galactopyranoside (0.009 g, 0.032 mmol) and 2,2′-bipyridine (0.014 g, 0.091 mmol) were dissolved in DMSO (10 mL) and then the solution was degassed by bubbling nitrogen for 20 min. Cu(I)Br (0.006 g, 0.04 mmol) was added into the mixture under nitrogen. The resulting solution was stirred at ambient temperature for 3 days, followed by addition of 50 mL of deionized water. The solution was bubbled with air for 6 h before transferred into a dialysis tube (NMWCO 2,000 Da). The solution was dialyzed against water for 2 days, followed by freeze-drying to give the glycopolymer as a white solid.

### Quantitative Precipitation Assay

This assay followed a modified procedure.^21^,^25^ Con A was dissolved in the HBS buffer (0.10 M HEPES, 0.9 M NaCl, 1 mM MgCl_2_, 1 mM CaCl_2_, and 1 mM MnCl_2_, pH 7.4) to make fresh stock solution and the concentration was 60 μM (assuming Con A tetramers with a molecular weight of 106 kDa). Glycopolymer solutions in HBS buffer were also prepared with a series of different concentration. Then Con A solution and the glycopolymer solution were mixed (1:1, v/v) energetically and incubated for 5 h at 22°C. So the final concentration of Con A was 30 μM. White precipitates were separated from solution by centrifugation at 5000 × *g* for 2 min, followed by removal of the supernatants very carefully using pipette. Then the pellets were resuspended in cold buffer again. These washing steps were repeated twice. After removal of the supernatants, the precipitates were dissolved in a water solution of methyl-α-d-mannopyranoside (1 mL, 1 M). With complete dissolution, the Con A content was determined by measuring the absorbance at 280 nm.

### Turbidimetry Assay

This was carried out following a previously described procedure by Kiessling, et al.^21^ Con A was fully dissolved in HBS buffer (∼1 mg/mL). The exact concentration of Con A was determined by measuring the absorbance at 280 nm [*A*_280_ = 1.37 × (mg/mL Con A)]. The solution was then diluted to 1 µM. After addition of 0.50 mL glycopolymer (50 µM) into a dry polycarbonate cuvette (2 mL, 1 cm path length), the cuvette was placed in the UV spectrometer. Following addition of 0.50 mL of the diluted Con A solution into the cuvette via a pipette the absorbance of the mixture was quickly recorded at 420 nm for 10 min every 0.12 s. The relative rate of interaction was determined by a linear fit of the steepest portion of the initial aggregation. Each experiment was repeated three times.

### Reversal Aggregation Assay

As previously described,^25^ following the turbidity measurement, the absorbance *A*_420_ of the solution after 2 h at ambient temperature was recorded as *A*_420_(*t* = 0). Subsequently, 0.1 mL methyl-α-d-mannopyranoside (54 mM) in HBS buffer solution was added to the cuvette. The mixed solution was quickly placed in the spectrometer and the absorbance at 420 nm was recorded for 10 min. *A*_420_(*t* = 10) was calculated as an average of the last 10 s of each run. The percent change in absorbance was determined as [*A*_420_(*t* = 0) − *A*_420_(*t* = 10)]/*A*_420_(*t* = 0).

### Inhibitory Potency Assay

Con A was dissolved in HBS buffer to make fresh stock solution and the concentration was 5 μM (assuming Con A tetramers with a molecular weight of 106 kDa). The stock solution of glycopolymer in HBS buffer was also prepared (5 μM). The glycopolymer solution (0.25 mL) and methyl-α-d-mannopyranoside (0.05 mL) of different concentration were mixed together, followed by addition of Con A solution (0.25 mL). The solution was mixed energetically and incubated for 5 h at 22°C and then the absorbance of the solution at 420 nm was measured.

### ConA-FITC Mannan Fluorescence Assay

A fluorescence absorption assay was used to assess the inhibition of the glycopolymers towards ConA. Microtitre plates were incubated for 16 h with 180 µL of 1 mg/mL mannan dissolved in phosphate-buffered saline (PBS) per well. Unattached mannan was removed by washing with PBS. Polymer solutions were made up as serial dilutions from 100 to 0.8 µg/mL in HBS. Twenty microliter of 0.3 mg/mL ConA-FITC in PBS was added to each polymer solution to result in 0.05 mM ConA-FITC. Hundred microliter of the polymer ConA-FITC solutions were added to the mannan surfaces and incubated at 37 °C for 30 min. Fluorescence was measured at excitation/emission wavelengths of 485/528 nm. All experiments were carried out in triplicate for a given polymer.

## RESULTS AND DISCUSSION

### Influence of Polymer Chain Length

**FIGURE 2:**
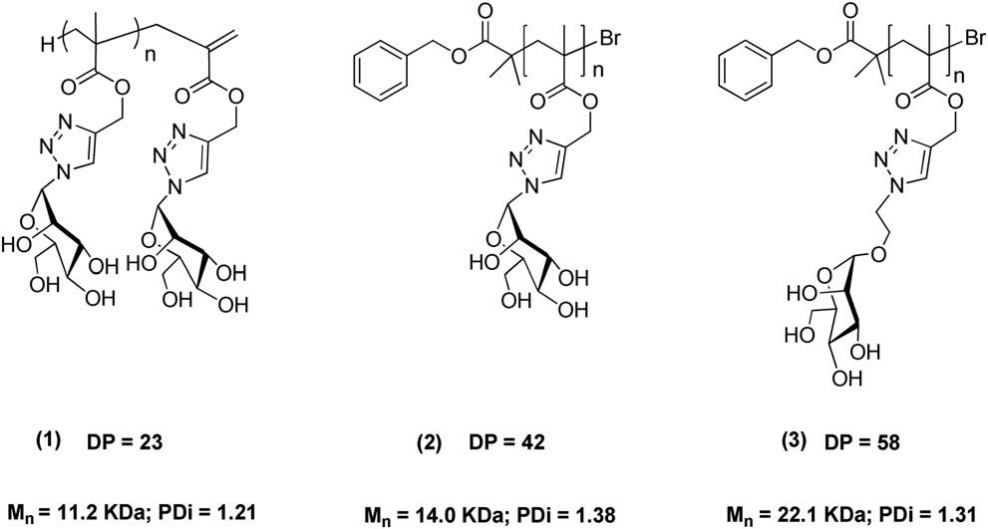
Mannose-containing glycopolymers with different chain lengths (degree of polymerization).

The chain length of glycopolymers is an important factor that influences lectin clustering.^21^ Thus, first we compared the binding properties of the glycopolymers of different chain lengths. The molecular weights and polydispersities of the glycopolymers are reported in Figure 2. Using the same monomer, glycopolymer **1** (DP = 23) was synthesized by catalytic chain transfer polymerization and CuAAC click reaction with α-azido-d-mannose, glycopolymer **2** (DP = 42) by living radical polymerization and CuAAC click reaction with α-azido-D-mannose, and glycopolymer **3** (DP = 58) was prepared by living radical polymerization and CuAAC click reaction with 2′-azidoethyl-O-α-d-mannopyranoside.

Con A was employed as a model lectin as it is structurally similar to many animal and bacterial lectins in cell communication events.^40^,^41^ It exists as a homotetramer at neutral pH with four identical binding units which can bind specifically to α-linked mannopyranosides and glucopyranosides. Con A is an excellent model for the lectin-glycopolymer interaction as it can be clustered by various multivalent ligands.^28^,^42^ As a single assay often elucidates only one aspect of the lectin-glycopolymer clustering, five assays were employed to fully explore the contribution of chain length to the inhibition and clustering of Con A. The results from quantitative precipitation, turbidimetry, inhibitory potency assays, ConA-FITC mannan fluorescence assays, and reversal aggregation assays are reported in Table 1. The stoichiometry of the glycopolymer-Con A conjugates (Con A units/polymer chain), the rate of the cluster formation [*k*_i_ (AU/min)], the inhibitory potency of these multivalent ligands [MIC50 (Mm) and IC50 (μM)], and the stability of the glycopolymer-Con A turbidity were investigated.

**TABLE 1:** The Results from Five Different Assays for the Investigation of Chain Length Influence

Mannose Glycopolymer	DP	Con A units/Polymer Chain	*k*_i_ (AU/min)	MIC_50_ (mM)	IC_50_ (μM)	[*A*_420_ (*t* = 0) − *A*_420_(*t* = 10)]/ *A*_420_ (*t* = 0) (%)
**1**	23	7	1.31	38.5	8.14	73.48
**2**	42	10	3.75	43.3	9.47	71.48
**3**	58	10	3.76	78	1.13	33.38

Glycopolymer **1** was poor at promoting receptor clustering and also possessed the least potent activity. The conjugation of **1** with Con A was very weak toward the disruption of the competitive ligand αMeMan indicating that a DP of 23 is too low for optimum binding. The stoichiometry of the Con A-glycopolymer complex and the rate of cluster formation were the same for glycopolymers **2** and **3**, although **2** was less effective and the resulting cluster was less stable than **3** relative to the monovalent ligand methyl-α-d-mannopyranoside. DP = 58 shows some benefits over DP = 42 but there is less difference between DP = 42 and 58. Thus, there seems to be less benefit in increasing the chain length after a certain length. Overall, glycopolymer **3** with DP = 58 showed to be the most effective in clustering Con A. Each polymer chain of **3** bound the most Con A units, the rate of the clustering was the highest and the affinity of this polymer to Con A was the strongest.

#### Synthesis of Glycopolymers

**SCHEME 1:**
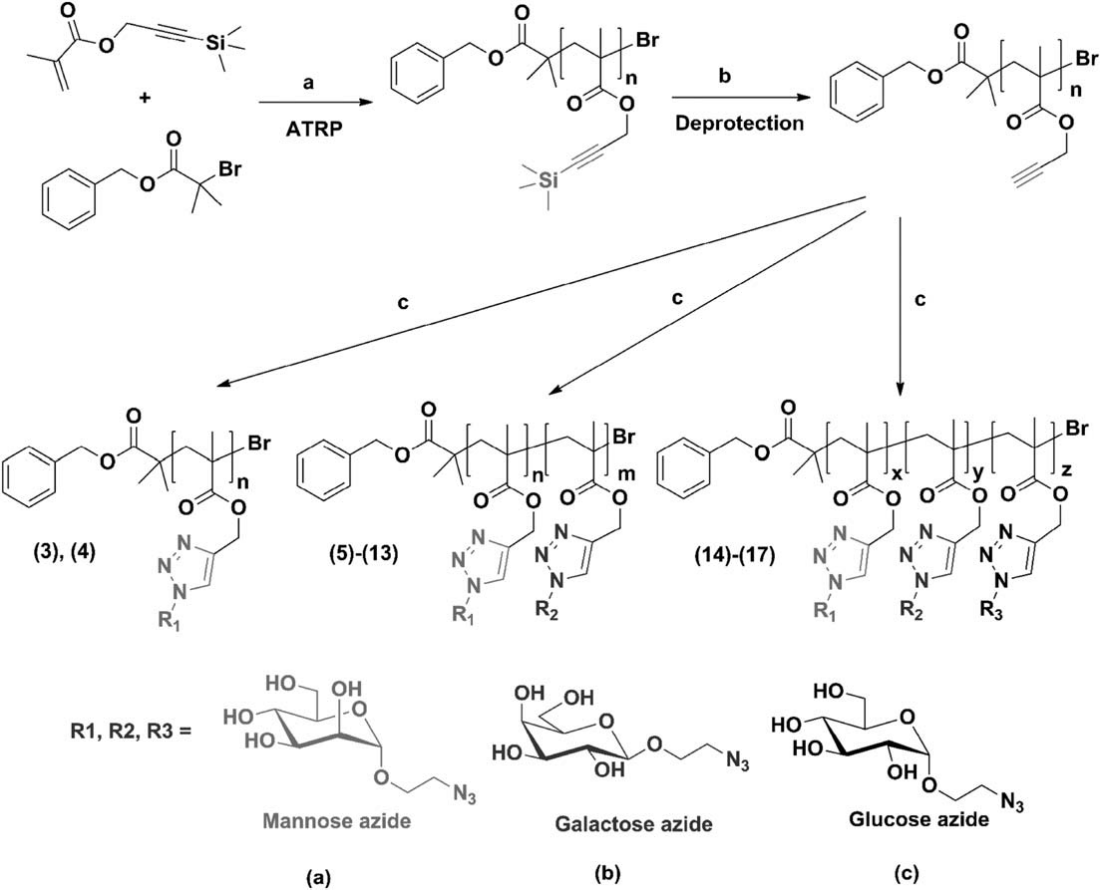
Synthesis of glycopolymers. (a) *N*-(ethyl)−2-pyridylmethanimine/Cu(I)Br, toluene, 70°C; (b) TBAF and acetic acid, THF; (c) RN_3_, CuBr, bipyridine, and Et_3_N.

As the mannose glycopolymer **3** with DP = 58 was the most effective in clustering Con A tetramers, to investigate the influence of different epitopes of various densities a library of well-defined glycopolymers were prepared via CuAAC by simultaneously attaching different sugar moieties (mannose, galactose, or glucose) to the same alkyne-containing polymer scaffolds, Scheme 1.

The monomer TMS-protected propargyl methacrylate was prepared from commercially available 3-trimethylsilylpropyn-1-ol and methacryloyl chloride. The polymerization was catalyzed by a Cu(I)Br/*N*-(ethyl)−2-pyridylmethanimine system.^43^ The alkyne-containing polymers were made by removal of the TMS protecting groups using TBAF with acetic acid as buffering agent. The clickable homopolymer was used as the precursor polymers. By clicking different ratios of 2′-azidoethyl-O-α-d-mannopyranoside **(a)** 2′-azidoethyl-O-β-d-galactopyranoside **(b)** and 2′-azidoethyl-O-α-d-glucopyranoside **(c)** onto the same polymer backbone via CuAAC, a library of glycopolymers were prepared, Table 2.

**TABLE 2:** List of Glycopolymers used in This Study

Entry	Mannoside (%)	Galactoside (%)	Glucoside (%)	*M*_w_/*M*_n_^a^	*M*_n_ (kDa)^b^
(3)	100	0	0	1.31	22.1
(4)	0	100	0	1.29	22.1
(5)	75	25	0	1.34	22.5
(6)	50	50	0	1.33	22.2
(7)	25	75	0	1.31	22.1
(8)	0	25	75	1.31	22.2
(9)	0	50	50	1.34	22.3
(10)	0	75	25	1.32	22.1
(11)	75	0	25	1.31	22.0
(12)	50	0	50	1.35	22.0
(13)	25	0	75	1.30	22.1
(14)	50	25	25	1.32	22.1
(15)	33	33	33	1.31	22.2
(16)	25	25	50	1.30	22.0
(17)	25	50	25	1.32	22.1

a Obtained by SEC analysis using DMF as eluent.

b Obtained by ^1^H NMR.

### Interactions of Glycopolymers with Con A

#### Stoichiometry of the Glycopolymer–Con A Conjugates

**FIGURE 3:**
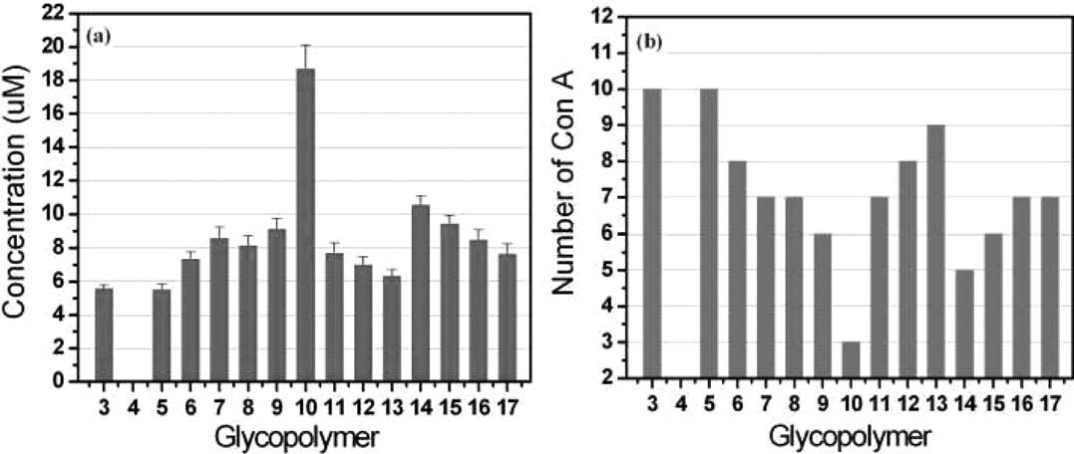
Quantitative precipitation results: (a) Concentration required for half-maximal precipitation. The error bars represent the standard deviation. (b) The number of Con A tetramers bound per glycopolymeric chain.

To investigate the capability of the glycopolymer chain to bind Con A tetramers, quantitative precipitation assays were employed.^44^ The concentrations of glycopolymer required to precipitate Con A from the solution of the same concentration were used to assess the glycopolymer concentration required for half-maximal precipitation of Con A, Figure 3(a). These results indicated the numbers of Con A tetramers bound to each glycopolymeric chain, Figure 3(b).

The results show that the mannose glycopolymer **3** binds the most Con A among all of the glycopolymers while **4** containing only pendant galactose cannot precipitate Con A. The mannose-galactose glycopolymers **5**, **6**, **7** bind more Con A tetramers than the glucose-galactose glycopolymers **8**, **9**, **10** with the same epitope density. These results from glycopolymers agree with the binding properties of the relative monosaccharaides to Con A. The densities of binding elements of these glycopolymers influence the clustering of Con A, however, this effect is decreasing as the densities increase > 75%. Comparing the number of Con A tetramers bound by the mannose-glucose glycopolymers **11**, **12**, **13**, we can conclude that the binding capability was greatly enhanced by grafting mannose instead of galactose to the polymeric backbone containing glucose. However, for all of the glycopolymers containing both mannose and glucose, glycopolymer **11** to **17**, mannose density is the dominant control factor on the binding capability. As small mannose density as 25%, the corresponding glycopolymers bind more Con A. Thus, both the nature and the density of pendant sugars have effect on the stoichiometry of the clustering complex. However, the homo-mannose glycopolymer **3** and the mannose-galactose glycopolymer **5** are most effective at binding copies of Con A per polymeric chain.

#### The Rate of Glycopolymer–Con A Clustering

**FIGURE 4:**
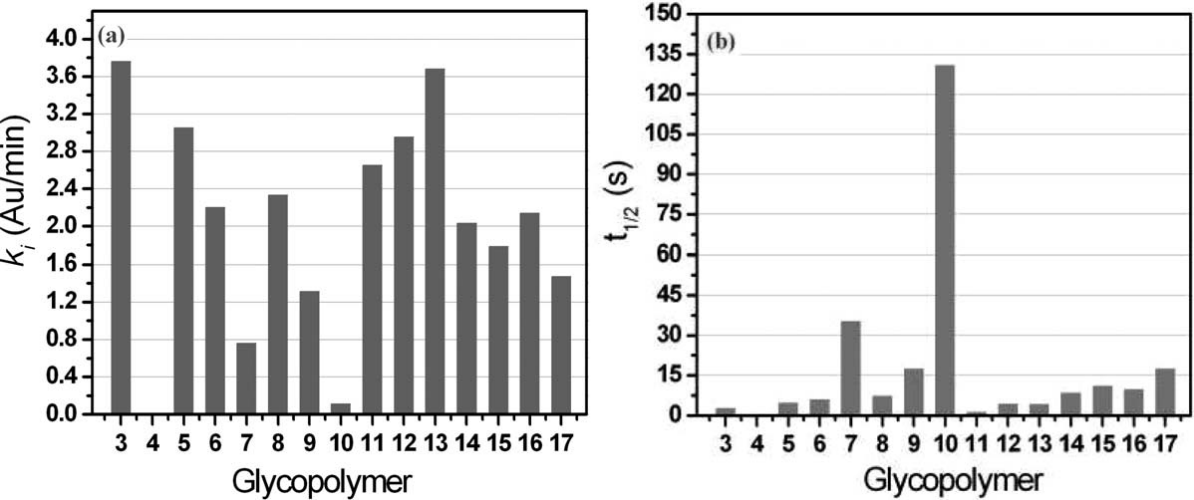
Results of turbidimetry experiments: (a) The initial rate of the clustering. (b) The time for half-maximal precipitation.

The rate of receptors clustering on the cell surface determine many signaling events, which can vary from seconds to hours.^45^–^47^ To monitor the clustering rate of the glycopolymer–Con A interaction in real time, turbidimetry experiments measuring the absorbance at 420 nm with time were carried out. A linear fit to the initial part of the curve was used to determine the rate of the clustering, which was expressed as arbitrary units per minute (AU/min), Figure 4(a). The endpoint of the curve was used to calculate the time for half-maximal precipitation of Con A, Figure 4(b).

The results reveal that glycopolymers **3**, **5**, **6**, **7** containing mannose moieties more rapidly initiated the clustering of Con A relative to glycopolymers **8**, **9**, **10** with pendant glucose of the same epitope density. Galactose moieties are the most effective sugar in regulating the rate of clustering. For glycopolymers **11**, **12**, **13** containing both mannose and glucose moieties, less mannose densities, faster the rate of the clustering is. These tendencies are confirmed by the mannose-galactose-glucose glycopolymers **14–17**. Most of the glycopolymers containing mannose moieties with no or low galactose density can promote the precipitation very rapidly (*t*_1/2_ < 10 s). Evidently, the precipitation of Con A by **10** is very slow (*t*_1/2_ = 130 s).

#### Inhibitory Potency of These Glycopolymers

**FIGURE 5:**
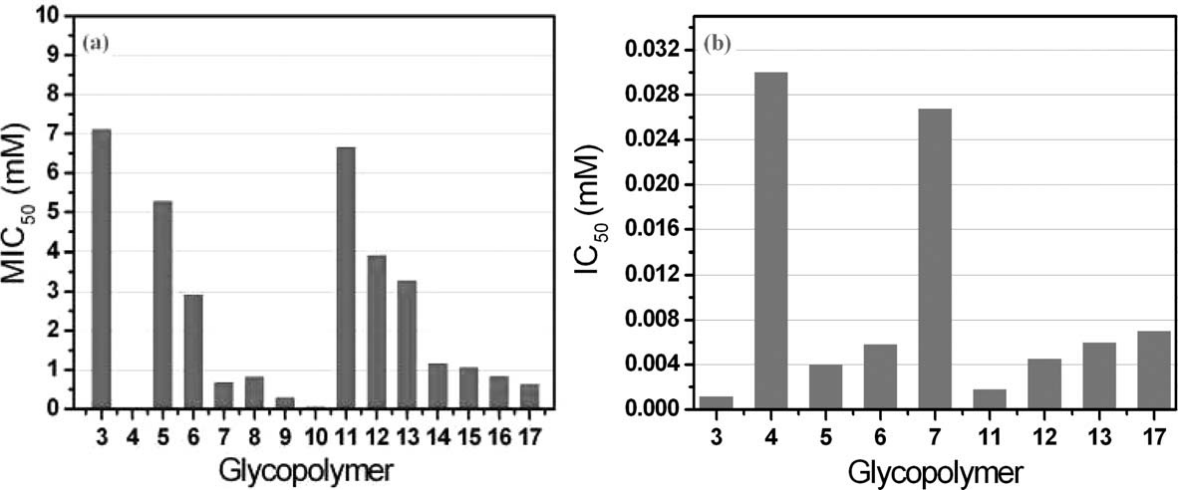
Results of inhibitory potency assays: (a) The MIC_50_ values of αMeMan for all the glycopolymers. (b) The IC_50_ values of the mannose-containing glycopolymers.

The inhibitory potency of multivalent ligands is a very important factor for them to be used to identify potent inhibitors.^28^ Assays were performed by adding Con A to the mixture of glycopolymer and methyl-α-D-mannopyranoside (αMeMan) to measure the absorbance at 420 nm of the resulting turbidity. Through varying the concentration of αMeMan, the minimum inhibitory concentration for half-maximum precipitation (MIC_50_) values of αMeMan were determined, Figure 5(a). Comparing the MIC_50_ values of αMeMan for all the glycopolymers, we can see that the density of mannose residues is the main factor in influencing on the potency of all the multivalent ligands and that glycopolymers with mannose moieties are much better inhibitors of Con A than those of the same binding-element concentration but with pendant glucose and galactose. The inhibitory potencies of mannose-containing glycopolymers decrease by adding glucose or galactose moieties onto the glycopolymeric backbone. Mannose glycopolymer **3** and the mannose-glucose glycopolymer **11** are the most effective inhibitors of Con A. These trends are further confirmed by the results obtained from the ConA-FITC mannan fluorescence assays. The inhibitory concentration for half-maximum precipitation (IC_50_) of Con A by mannose-containing glycopolymer against mannan depends mainly on the ratio of the mannose moieties in the glycopolymers, Figure 5(b).

#### The Stability of Glycopolymer–Con A Cluster

**FIGURE 6:**
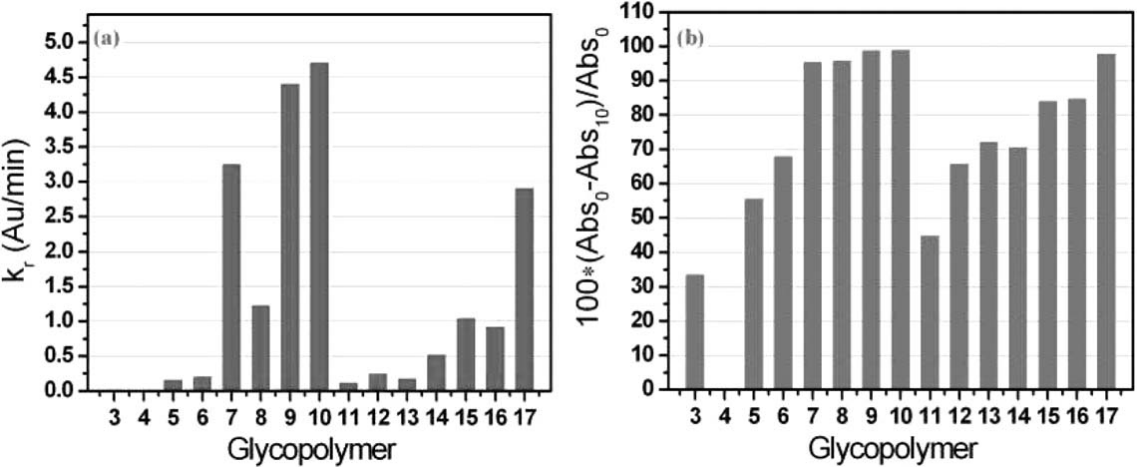
Results of reversal aggregation assays: (a) the rate of the reverse interaction between the turbidity and αMeMan. (b) The percent change of the turbidity after 10 min with the addition of αMeMan solution.

As the lectin-carbohydrate interaction is reversible, the inhibitory potency assays measured the ability of these multivalent ligands in the kinetic competition with αMeMan for clustering Con A. To investigate the stability of the glycopolymer-Con A complexes, reversal aggregation assays were employed.^25^ By addition of monovalent ligand αMeMan of the same concentration into the turbidity solution, the rate of the reverse interaction was determined by a linear fit of the steepest portion of the data, Figure 6(a). The percent change of the turbidity after 10 min was calculated using [*A*_420_(*t* = 0) − *A*_420_(*t* = 10)]/*A*_420_(*t* = 0), Figure 6(b). Mannose glycopolymer **3** is very stable and the reverse interaction is very slow. The results show that the lectin conjugates induced by glucose residues of glycopolymers **7**, **8**, **9** quickly interact with the monovalent sugar and the turbidity almost disappears after 10 min. A comparison of all of the multivalent ligands, the influence of galactose on the stability is obvious only when the pendant galactose density is >50%. The density of mannose moieties in the multivalent ligands is the key factor, which determines the stability of the resulting lectin-glycopolymer cluster.

#### Comparison of Glycopolymers with Respect to Different Aspects of the Glycopolymer–Con A Interaction

**FIGURE 7:**
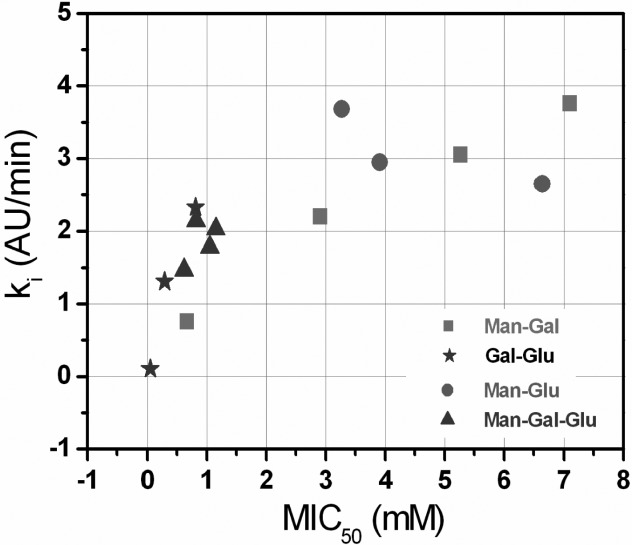
The rate of clustering and inhibitory potency for all of the multivalent ligands.

Taking the rate of clustering and the inhibitory potency into consideration simultaneously, it is very interesting that the difference of these glycopolymers in clustering Con A tetramers is quite obvious (Fig. 7). Overall, mannose-containing glycopolymers are better than the glucose-containing glycopolymers and the mannose glycopolymer **3** is the most effective inhibitor among all the multivalent ligands. However, addition of glucose moieties in place of galactose can slightly change the rate of clustering or the potency of the obtained macromolecular ligand. The clustering rates and inhibitory potencies of the mannose-galactose-glucose glycopolymers **14–17** are similar to **6** and **7**. With the diversity of the rate and the potency, different multivalent ligands are provided to be chosen for specific functions.

## CONCLUSIONS

As the mannose glycopolymer **3 (**DP = 58) was the most effective in clustering Con A tetramers in the investigation of the influence of chain lengths, a series of glycopolymers were prepared by the combination of living radical polymerization and CuAAC using the same alkyne-containing precursor polymers to study the influence of different epitopes of various densities. By post-functionalization of the precursor polymers via clicking different sugar azides onto the polymeric backbone, the well-defined glycopolymers were generated featuring the same macromolecular properties (architecture, polydispersity, valency, polarity, etc.) with difference only in the densities of different sugars (mannose, galactose, and glucose). This synthetic strategy is significantly important for the investigation of the influence of various pendant epitopes on the lectin-multivalent interactions.

Employing five different efficient assays, quantitative precipitation, turbidimetry, inhibitory potency assay, fluorescence assay, and reversal aggregation assay, allowed for the exploration of the behavior of the 15 different multivalent ligands in clustering receptors using Con A as the model lectin. The stoichiometry of the glycopolymer-Con A conjugates, the rate of the cluster formation, the inhibitory potency of these multivalent ligands and the stability of the glycopolymer-Con A turbidity were investigated.

The glycopolymer **3,** fully substituted with one mannose residue per repeat unit, was the most efficient multivalent ligand for clustering Con A in all of the experiments. The mannose density is the dominant factor for the binding stoichiometry, the rate of binding, the potency and the stability of Con A clustering. However, the galactose residues of different densities are effective in regulating the rate of cluster formation. Although the glucose-induced clusters are not very stable toward the disruption caused by the competitive monovalent ligand methyl-α-d-mannopyranoside, glucose moieties of the glycopolymers are important for the stoichiometry and the rate of the interactions by coworking with mannose residues.

Thus, the diversities of binding properties contributed by different clustering parameters can make it possible to define the structures of the multivalent ligands and densities of binding epitopes for specific functions in the lectin-ligand interactions. These conclusions can be employed to develop new glycopolymeric drugs and therapeutic agents and to assess the mechanisms by which they work.
