# Supplementary material for: A Detailed Study on Understanding Glycopolymer Library and Con A Interactions
Source: J Polym Sci A Polym Chem. 2013 Mar 13;51(12):2588–97. doi: 10.1002/pola.26646 (PMC3677416; doi:10.1002/pola.26646)
Supplement: Supplementary file 1 [file pola0051-2588-SD1.doc]

Supporting information for

Multivalent ligands with different sugars of various densities tailor-made for the receptor-ligand interaction

Yanzi Gou,^*^ Jin Geng,^b^ Sarah-jane Richards,^c^ James Burns,^c^ Mathew I. Gibson,^c^ C. Remzi Becer^c^ and D. M. Haddleton^c*^

**a**: Science and Technology on Advanced Ceramic Fibers and Composites Laboratory, National University of Defence Technology, Changsha 410073, China

**b**: Faculty of Engineering, University of Bristol, Clifton BS8 1TR, United Kingdom
**c**: Department of Chemistry, University of Warwick, Coventry, CV4 7AL, United Kingdom

Corresponding author:

Prof. D. M. Haddleton : [D.M.Haddleton@warwick.ac.uk](mailto:D.M.Haddleton@warwick.ac.uk)

Dr. Yanzi Gou : [Y.Gou2012@hotmail.com](mailto:Y.Gou2012@hotmail.com)

**Quantitative precipitation** The assay was followed a modified procedure.^1-2^ Con A was dissolved in the HBS buffer (0.10 M HEPES, 0.9 M NaCl, 1 mM MgCl_2_, 1 mM CaCl_2_ and 1 mM MnCl_2_, pH 7.4) to make fresh stock solution and the concentration was 60 μM (assuming Con A tetramers with a molecular weight of 106 kDa). Glycopolymer solutions in HBS buffer were also prepared with a series of different concentration. Then Con A solution and the glycopolymer solution were mixed (1:1, v/v) energetically and incubated for 5 hours at 22 °C. So the final concentration of Con A was 30 μM. White precipitates were separated from solution by centrifugation at 5000 × g for 2 minutes, followed by removal of the supernatants very carefully using pipette. Then the pellets were resuspended in cold buffer again. These washing steps were repeated twice. After removal of the supernatants, the precipitates were dissolved in a water solution of methyl-α-D-mannopyranoside (1 mL, 1 M). With complete dissolution, the Con A content was determined by measuring the absorbance at 280 nm.


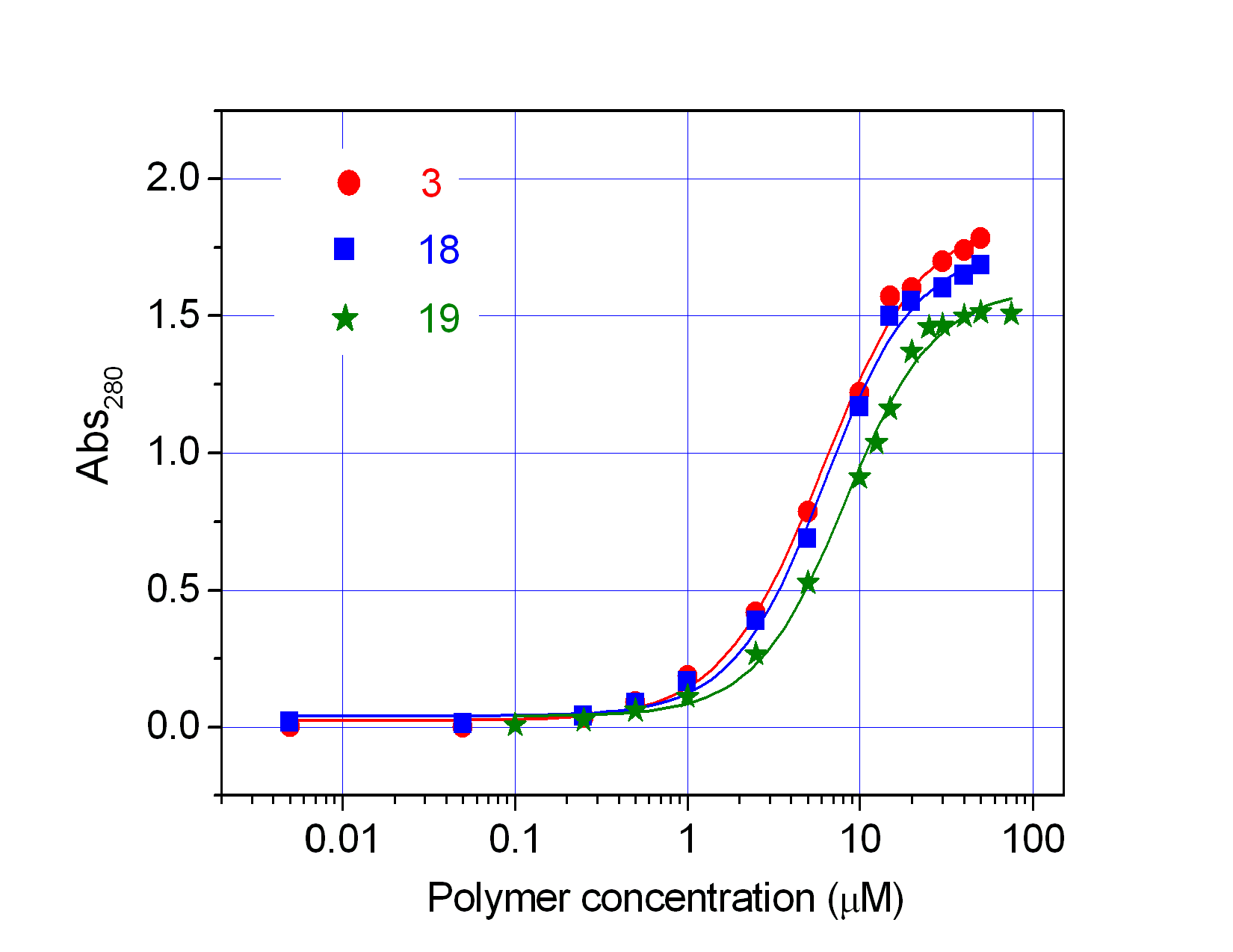












**Fig. 1** Sigmoidal curves fitted to quantitative precipitation data for all the glycopolymers.

**Turbidimetry** It was carried out via a previously described procedure by Kiessling, et al.Error: Reference source not found Con A was fully dissolved in HBS buffer (approximately 1 mg/mL). The exact concentration of Con A was determined by measuring the absorbance at 280 nm (A_280_ = 1.37 × [mg/mL Con A]). The solution was then diluted to 1 µM. After addition of 0.50 mL glycopolymer (50 µM) into a dry polycarbonate cuvette (2 mL, 1 cm pathlength), the cuvette was placed in the UV spectrometer. By adding 0.50 mL of the diluted Con A solution into the cuvette via a pipette, the absorbance of the mixture was quickly recorded at 420 nm for 10 min every 0.12 s. The relative rate of interaction was determined by a linear fit of the steepest portion of the initial aggregation. Each experiment was repeated 3 times.


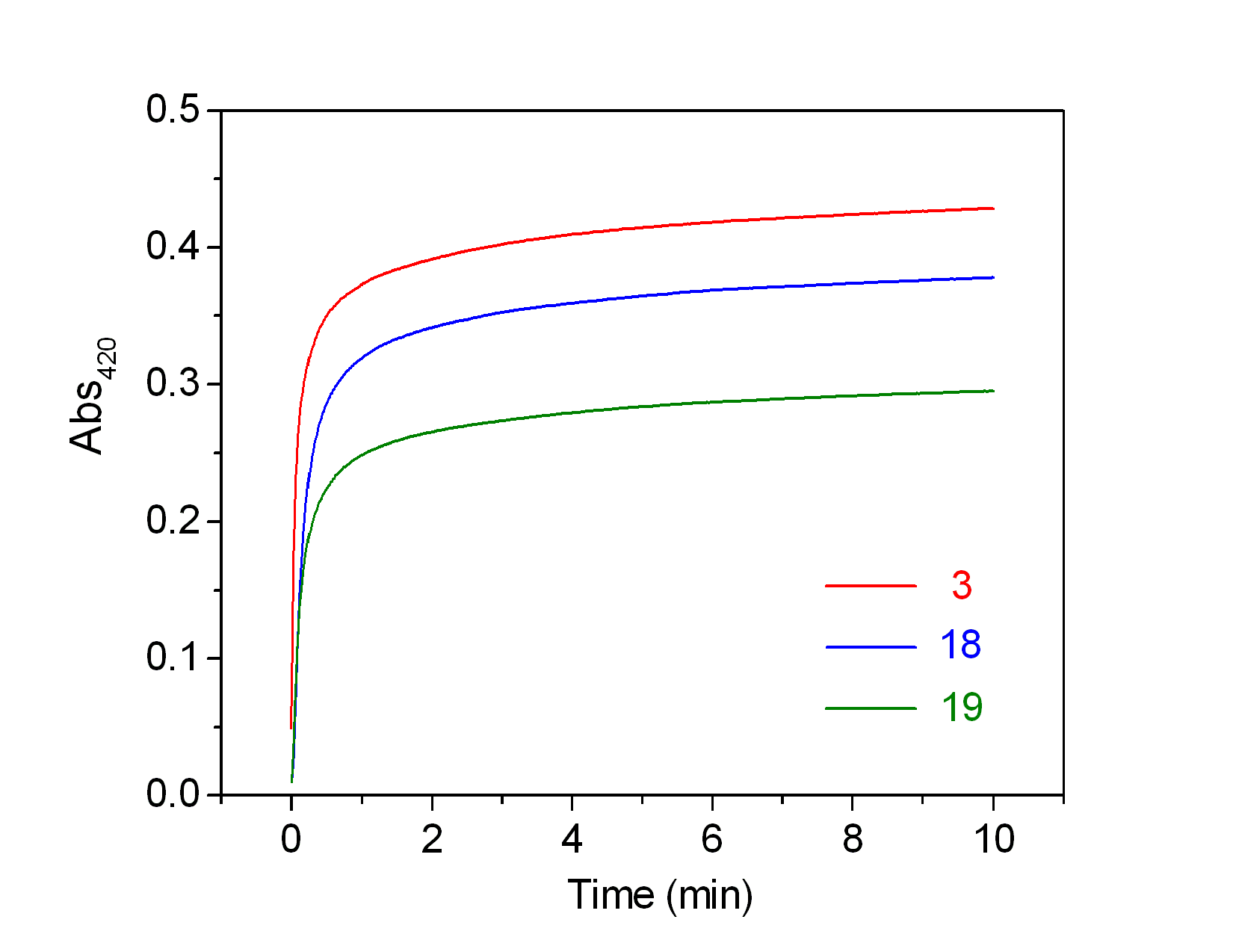












**Fig.2** The results of turbidimetry for all the glycopolymers.
