# Supplementary material for: A Detailed Study on Understanding Glycopolymer Library and Con A Interactions
Source: J Polym Sci A Polym Chem. 2013 Mar 13;51(12):2588–97. doi: 10.1002/pola.26646 (PMC3677416; doi:10.1002/pola.26646)
Supplement: Supplementary file 2 [file pola0051-2588-SD2.doc]

**Reversal aggregation assays** As previously described,Error: Reference source not found following the turbidity measurement, the absorbance A_420_ of the solution after 2 hours at room temperature was recorded as A_420_(t=0). Then 0.1 mL methyl-α-D-mannopyranoside (54 mM) in HBS buffer solution was added to the cuvette. The mixed solution was quickly placed in the spectrometer and the absorbance at 420 nm was recorded for 10 minutes. A_420_(t=10) was calculated as an average of the last 10 seconds of each run. The percent change in absorbance was determined as (A_420_(t=0)-A_420_(t=10))/ A_420_(t=0).












**Fig.3** The results of reversal aggregation assays for all the glycopolymers.

**Inhibitory potency assay** Con A was dissolved in HBS buffer to make fresh stock solution and the concentration was 5 μM (assuming Con A tetramers with a molecular weight of 106 kDa). The stock solution of Glycopolymer in HBS buffer was also prepared (5 μM). The glycopolymer solution (0.25 mL) and methyl-α-D-mannopyranoside (0.05 mL) of different concentration were mixed together, followed by addition of Con A solution (0.25 mL). The solution was mixed energetically and incubated for 5 hours at 22 °C and then the absorbance of the solution at 420 nm was measured.


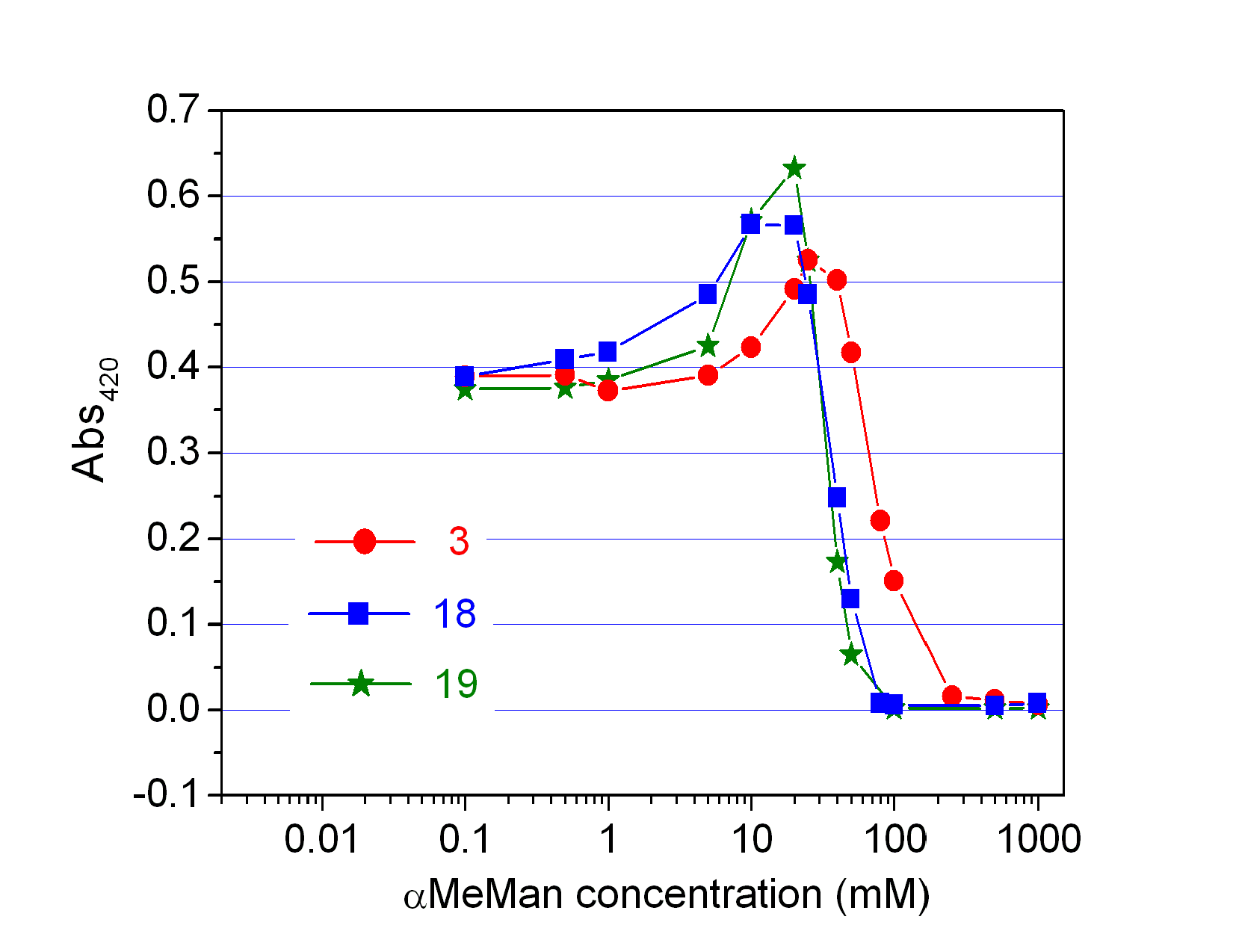


**

**




**

**

**Fig.4** The results of inhibitory potency assays for all the glycopolymers.

**References**

**Error: Reference source not found**
